# A Common Cold

**Published:** 1870-03

**Authors:** 


### HALL'S JOURNAL OF HEALTH.

VOL. XVII. MARCH, 1870. NO. 3.

### A COMMON COLD,

In a true practical popular manner, has never been described since the world began, and outside of the profession there is not a man in a million who has a clear philosophical idea of what it is, or the manner of its action on the human system.

It is just as impossible for a bad cold to cause (originate) consumption as it would be for a barrel of powder to blow up a house; and yet nine out of ten, even of educated persons, consider it as among the undeniable facts that a cold has an unmistakable influence in bringing on common consumption of the lungs in the sense of originating it, and this in face of the fact that every one has a cold several times every year during a whole life-time and yet four-fifths of the people die of diseases which are not consumptive. If a cold causes consumption, why is it that four-fifths of grown persons,—yes, nine-tenths of them, have had colds hundreds of times in a life-time, yet do not have consumption. The truth is that colds have no power to originate consumptive disease; when the seeds of the malady are already in the lungs, a bad cold may develop it, as a spark will cause a barrel of powder to blow up a house, if brought in contact with it, not otherwise.

A cold is never cured; and yet we have millions of remedies published every year which will cure a cold. A cold cures itself. That is, a man gets well of a cold as he gets well of small-pox, but we do not remember ever to have heard of a man being cured of small-pox; this fearful malady runs its course, and the man gradually regains his health. So a common cold, fairly seated, will run its average course of two weeks, and then the man is as well as he ever was. One who is of a vigorous constitution will "throw off" the cold sooner than one who is frail; but even the feeble would "get rid" of the cold much sooner than is commonly done if this cold was not renewed; but multitudes have found that when they have a cold, the least thing in the world renews it, and in consequence of these frequent renewals it hangs on, and hangs on for weeks and months, sometimes for a whole winter, although it is always the case that summer colds, colds taken in weather comparatively warm, are more difficult to get rid of. In a warm winter, for example, or when the weather is almost too warm to have a fire, it is proverbial that almost everybody has a cold, and more persons die than when the weather is steadily cold. One main reason of this is, we are dressed warm; slight exercise or work excites perspiration, and this being checked in a great variety of ways gives a cold, which "settling" on some weak or diseased part, becomes the immediate, exciting cause of death; hence a very frequent remark in reference to the death of friends, "it seemed to be but a little cold at first."

A cold may be cut short; its usual course of ten days or two weeks is often abbreviated by the accidental intervention of some other form of disease. For example, a large boil may form; this attracts disease from the lungs and the cold is no longer heard of; the same result follows an attack of diarrhœa, bilious, or otherwise; in this case the cold is said "to run off through the bowels." If these conditions are artificially excited, if the bowels are made loose by medicines calculated to act upon them, the progress of the cold is shortened, provided in all cases that these things occur within thirty-six hours after the cold is taken; if fairly seated, it will take its course.

Most persons can tell in a few hours, or even minutes, when they have taken cold; when the slightest chill runs down the back, a cold has been taken; sneezing indicates that a cold has been taken, so does a running from the nose, not always, but often; if a person would go to bed, wrap up warm, drink hot teas abundantly and not eat any thing but the crust of bread, which is light, yet nutritious, and not tempting, the cold would be cut short in twenty-four hours; but nobody will follow such a direction, as there is always a vague impression that it may pass away without giving special trouble, which indeed is the case in many instances in the experience of each individual; still the safer plan would be to act as if the cold might be a very bad one, as much discomfort, actual sickness, and life itself would be often saved thereby.

The reason why there are so many things advised for curing colds, and with such overweening confidence, is simply this: when a cold is taken, the first thing thought of is to "take something" for it; if one thing fails, then another is tried, until perhaps a dozen different things have been experimented upon, meanwhile the cold has about run its course and is curing itself, and the remedy which happened to be in use at the time gets the credit of the cure, and the gratified individual "swears by" that remedy to the latest hour of life, never failing to recommend it with fanatical enthusiasm and confidence to every man, woman, and child who happens to cough in his presence.

When a person takes a cold the natural remedy would seem to be to take a warm, that is get warm and keep warm for every second of time, keep comfortably warm, even if it requires the thermometer to be kept at a hundred degrees, and maintain this temperature and remain in a well warmed and ventilated room until restored to usual health.

'FEED A COLD,"

And starve a fever, is the proverb, but it is most fallacious. From the moment a cold is fairly settled every atom of food taken lays the foundation for more cough and for the longer continuance of the cold. All know that a cold does not begin to get well until the "cough begins to loosen," that is until yellow matter is brought up from the lungs, as a boil never begins to get well until yellow matter is discharged from it; this yellow matter in both cases is made from the blood, the blood is made out of the food eaten, so the more food we eat while we have a cold, the more blood we make; and the more material for yellow matter, the more of such matter is made, and the more coughing does it require to bring it all away; until that is done, no one gets well of a cold.

### COUGH IS CURATIVE,

Both of consumption and of a bad cold, because it is the cough, incited by nature, which helps in both cases to clear the lungs of the yellow matter in them, and it is just as impossible for them to get well in either case until this yellow matter is all removed, as it is for a boil to get well until all its yellow matter is poured out of it. And yet the universal practice in consumption as well as in bad colds, is to want to be taking something to

### "CURE THE COUGH."

And whatever is swallowed and has the effect to lessen the cough is lauded to the skies with gratitude, as a great remedy; in the height of the enthusiasm, a very pronounced certificate is given; this is published in the papers, and spread before millions of readers in a month, but the death of the certifier within the month, follows not after the falsehood. A truer practice is to promote the cough, to loosen the phlegm, so as to make the cough more frequent and necessary, to raise it out of the way: nothing can be more true than that cough is curative, both of consumption and bad colds; it is nature's remedy.

